# Supplementary figures and images for: Heat Shock Factor 1 forms nuclear condensates and restructures the yeast genome before activating target genes
Source: eLife. 2024 Oct 15;12:RP92464. doi: 10.7554/eLife.92464 (PMC11479590; doi:10.7554/eLife.92464)

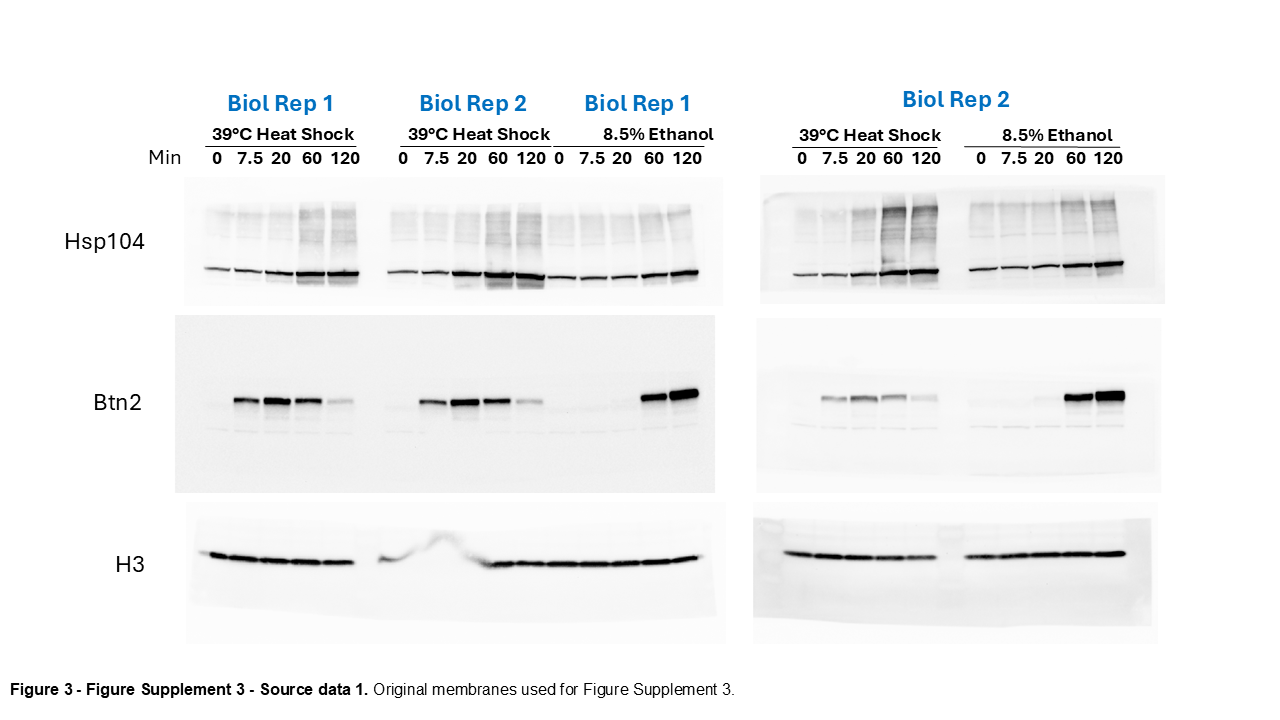

Supplement: Figure 3—figure supplement 3—source data 1. [file elife-92464-fig3-figsupp3-data1.tif]

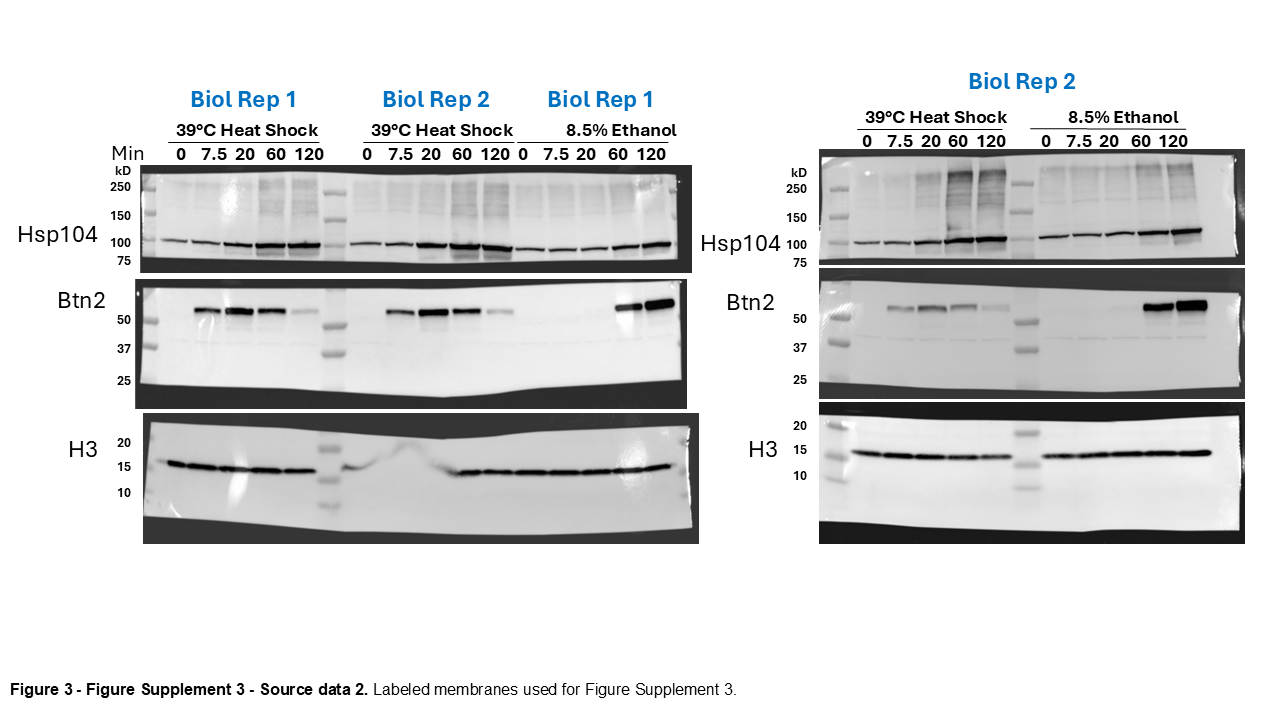

Supplement: Figure 3—figure supplement 3—source data 2. [file elife-92464-fig3-figsupp3-data2.tif]

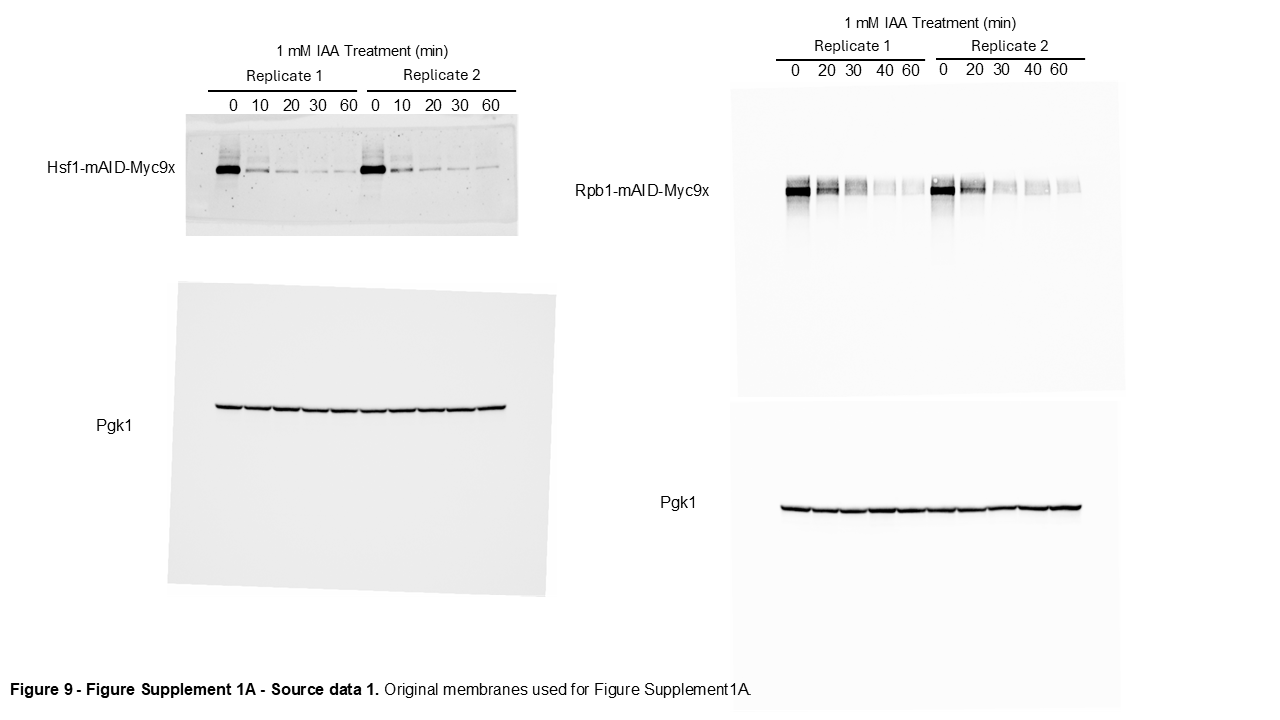

Supplement: Figure 9—figure supplement 1—source data 1. [file elife-92464-fig9-figsupp1-data1.tif]

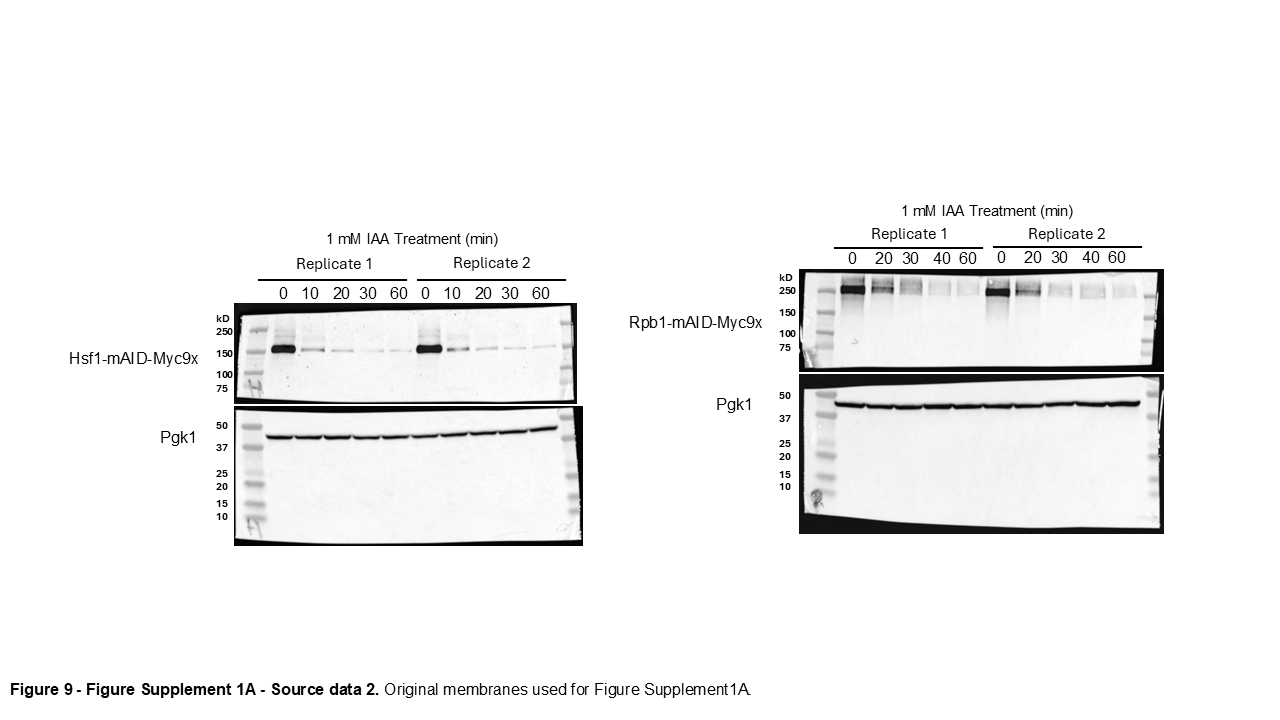

Supplement: Figure 9—figure supplement 1—source data 2. [file elife-92464-fig9-figsupp1-data2.tif]
